# Ethical use of artificial intelligence in education: proposed ethical competency framework for teachers

**DOI:** 10.3389/frai.2026.1833043

**Published:** 2026-08-03

**Authors:** Ibrahim Yaussef Alyoussef, Nisar Ahmed Dahri, Khadijah Amru Alhashmi, Waleed Mugahed Al-Rahmi, Paras Muzna, Fida Hussain Dahri

**Affiliations:** 1 Education Technology, Faculty of Education, King Faisal University, Al-Ahsa, Saudi Arabia; 2 Faculty of Educational Sciences and Technology (FEST), Universiti Teknologi Malaysia, Johor Bahru, Malaysia; 3 Sohar University, Sohar, Oman; 4 Department of Educational Policies, College of Education, Umm Al-Qura University, Mecca, Saudi Arabia; 5 Department of Management Information System, College of Business Administration, Dar Al Uloom University, Riyadh, Al Falah, Saudi Arabia; 6 Department of Education, University of Sindh, Jamshoro, Pakistan; 7 School of Computer Science and Technology, Harbin Institute of Technology, Harbin, China

**Keywords:** AI integration, artificial intelligence, education technology, ethical competencies, teachers

## Abstract

**Introduction:**

The rapid integration of Artificial Intelligence (AI) in education is transforming teaching, learning, and professional development through personalized instruction, adaptive learning, and data-driven educational practices. However, in Pakistan and Saudi Arabia, many teachers still lack the ethical awareness and practical competencies required for the responsible use of AI technologies. Challenges related to data privacy, algorithmic bias, transparency, accountability, and equitable access continue to hinder effective AI adoption in teacher education and classroom practice. This study proposes a contextualized ethical competency framework to support the responsible and effective integration of AI among teachers in both countries.

**Methods:**

An exploratory sequential mixed-methods design was employed, comprising a systematic literature review and a pilot implementation study involving 30 teachers from diverse educational levels and institutional contexts. The framework was developed through a qualitative synthesis of international AI ethics frameworks, responsible AI principles, and teacher competency literature. It was subsequently evaluated using quantitative measures and qualitative feedback collected from participating teachers.

**Results:**

The findings provide preliminary evidence that teachers perceived the proposed framework positively in terms of effectiveness (M = 3.73), usability (M = 3.63), and ethical compliance (M = 3.83). Qualitative findings further indicated that the framework enhanced ethical awareness, responsible AI use, personalized instructional practices, and AI-supported learning design. Participants also identified several implementation challenges, including limited prior AI knowledge, insufficient technical support, and the need for continuous professional development.

**Discussion and conclusion:**

This study contributes to the growing body of research on ethical AI integration in education by introducing a localized framework that combines ethical governance with practical AI integration competencies for teachers in Pakistan and Saudi Arabia. The findings underscore the importance of sustained teacher training, institutional readiness, ethical governance, and supportive policies to facilitate responsible AI adoption in education. Future research should conduct large-scale longitudinal studies to further validate the framework and evaluate its scalability, effectiveness, and long-term educational impact across diverse educational settings.

## 1 Introduction

Artificial Intelligence (AI) is rapidly transforming educational systems worldwide by reshaping teaching practices, learning environments, and institutional management processes. The integration of AI technologies into education has created new opportunities for personalized learning, adaptive instruction, intelligent assessment, and data-driven educational decision-making. AI-supported educational tools, including intelligent tutoring systems, virtual teaching assistants, automated assessment platforms, and learning analytics systems, are increasingly being used to enhance teaching effectiveness and improve student engagement (Yoo et al., 2022). Recent advancements in generative AI technologies, such as ChatGPT, Google Bard, and AI-based learning assistants, have further accelerated the adoption of AI-supported teaching and professional development practices across educational institutions (Dahri et al., 2024a).

AI-based educational applications can provide individualized feedback, adapt instructional content to students’ learning needs, and support immersive and interactive learning experiences (Srinivasa et al., 2022; Yilmaz and Yilmaz, 2023). In addition, AI technologies may assist teachers in lesson planning, classroom management, instructional design, automated grading, and administrative tasks, thereby reducing workload and supporting more flexible and student-centered learning environments (Al-Rahmi, 2026a; Anggarini et al., 2023). AI-supported virtual teaching simulations and intelligent feedback systems are also increasingly used within teacher professional development programs to strengthen pedagogical practices and digital teaching competencies. However, the effective use of AI technologies in education requires more than technical access alone. Teachers must develop practical AI integration skills alongside ethical awareness to ensure that AI-supported educational practices remain human-centered, transparent, inclusive, and pedagogically appropriate (Alwakid et al., 2025; UNESCO, 2024).

Despite the growing adoption of AI technologies in education, significant challenges remain regarding teachers’ preparedness for responsible AI integration, particularly within developing educational contexts such as Pakistan and Saudi Arabia. Existing studies have largely focused on technical AI adoption and digital competencies, while comparatively less attention has been given to the ethical competencies required for AI-supported classroom practices and teacher professional development. Many teachers continue to face difficulties in effectively integrating AI technologies due to limited technical proficiency, insufficient professional training, and lack of institutional support (Jonbekova, 2020; Younas, 2020). The rapid digital transformation experienced during and after the COVID-19 pandemic further highlighted the importance of resilient, technology-supported educational systems and the urgent need for AI-related teacher preparedness (kyzy Aijan and Sharma, n. d.).

At the same time, the rapid expansion of AI in education has raised several ethical concerns related to data privacy, algorithmic bias, transparency, accountability, misinformation, and unequal access to educational technologies (Al-Rahmi, 2026b,c; Farahani and Ghasemi, 2024; Khreisat et al., 2024). AI systems trained on large datasets may unintentionally reinforce existing educational and social inequalities if fairness, inclusiveness, and contextual diversity are not adequately considered during implementation. Ethical concerns related to human–AI interaction, power relations, automated decision-making, and the reliability of AI-generated educational content have therefore become increasingly important within teacher education and professional development programs (Tawafak et al., 2025; Filgueiras, 2023; Miao et al., 2021). Consequently, teachers are expected not only to use AI technologies effectively, but also to critically evaluate their ethical implications and ensure responsible implementation within classroom environments.

In Pakistan and Saudi Arabia, educational systems are undergoing rapid digital transformation through national initiatives aimed at strengthening educational modernization and technological innovation. Saudi Arabia’s Vision 2030 and Pakistan’s growing investment in digital education and smart learning technologies have accelerated interest in AI-supported educational systems and teacher professional development (Al-Rahmi et al., 2026; Al-Amri and Al-Abdullatif, 2024; Foroughi et al., 2023; Zubairi et al., 2021). However, empirical studies focusing specifically on teachers’ ethical AI competencies within these contexts remain limited. Existing regional studies primarily emphasize digital transformation, AI adoption readiness, and technological infrastructure, while ethical AI preparedness and responsible classroom integration remain underexplored changes (Al-Rahmi, 2025; Dahri et al., 2024a; Khan and Alamäki, 2023; Al-Maroof et al., 2025).

Globally, similar challenges have been observed in AI-integrated educational systems. China has introduced AI competency initiatives for teachers; however, several studies indicate that these frameworks place greater emphasis on technical AI literacy while comparatively less attention is given to ethical dimensions such as transparency, accountability, and human–AI collaboration within classrooms. In South Korea and Singapore, concerns regarding algorithmic bias, student data protection, and ethical governance of AI systems have received considerable scholarly attention. Finland’s AI education strategy, although highly advanced in terms of inclusiveness and digital innovation, still faces challenges regarding teacher preparedness and the broader societal implications of AI-supported learning environments (Al-Maroof et al., 2025; Alshaibani et al., 2025; Alfarsi et al., 2024a,b; Shin D., 2019; Shin D. D. H., 2019). These international experiences highlight the growing global need for ethical AI competency frameworks capable of supporting responsible, transparent, and human-centered AI integration within education.

Given these challenges, there is an urgent need to develop contextually relevant ethical competency frameworks that can support teachers in responsibly integrating AI technologies into educational environments. Such frameworks should combine practical AI integration competencies with ethical awareness related to transparency, fairness, accountability, inclusiveness, human-centered AI practices, and data privacy protection. This need is particularly important in Pakistan and Saudi Arabia, where AI-supported educational transformation is rapidly expanding while structured ethical AI competency models for teachers remain limited.

Therefore, this study aims to develop and preliminarily evaluate a contextualized ethical competency framework to support the responsible and effective integration of AI in teacher education and professional development within Pakistan and Saudi Arabia. Unlike existing international AI competency initiatives that primarily emphasize technical AI literacy or generalized ethical principles, the proposed framework integrates both practical AI integration skills and ethical governance dimensions tailored to the sociocultural, institutional, and educational realities of the two countries.

Specifically, this study seeks to:

- Investigate the major ethical challenges associated with AI integration in educational settings within Pakistan and Saudi Arabia.
- Identify the key ethical and practical competencies required for teachers to use AI technologies responsibly and effectively.
- Develop a contextualized ethical competency framework for AI-supported teaching and professional development.
- Conduct a preliminary pilot evaluation of the proposed framework among teachers from diverse educational contexts in Pakistan and Saudi Arabia while providing policy and professional development recommendations for sustainable and ethical AI integration in education.

## 2 Methodology

### 2.1 Research design

This study adopted an exploratory sequential mixed-methods research design to investigate the ethical integration of Artificial Intelligence (AI) in teacher education and professional development in Pakistan and Saudi Arabia. The exploratory sequential design was considered appropriate because the study initially explored ethical AI challenges and competency dimensions through a systematic literature review and conceptual analysis, followed by a pilot quantitative evaluation of the proposed framework among teachers. Mixed-methods research enables researchers to combine qualitative conceptual exploration with quantitative validation, thereby improving the depth, reliability, and practical applicability of findings (Creswell et al., 2003). The integration of qualitative and quantitative approaches has also been widely recommended in educational technology and AI-related educational research due to the multidimensional nature of ethical AI adoption in educational environments (Dahri et al., 2024a; Namatovu and Kyambade, 2025).

The qualitative phase focused on identifying key ethical concerns, AI integration competencies, and responsible AI practices from prior literature and international AI competency frameworks. The quantitative phase subsequently examined teachers’ perceptions regarding the usability, effectiveness, and ethical applicability of the proposed framework through a pilot implementation study. The integration of both phases provided a comprehensive understanding of AI-related ethical competencies required for teachers within the educational contexts of Pakistan and Saudi Arabia.

### 2.2 Systematic literature review procedure

A systematic literature review (SLR) was conducted in accordance with the PRISMA (Preferred Reporting Items for Systematic Reviews and Meta-Analyses) guidelines to ensure methodological rigor, transparency, and reproducibility (Page et al., 2021). The primary objective of the review was to identify existing ethical challenges, competency dimensions, and established artificial intelligence (AI) integration frameworks in the field of education, with particular emphasis on teacher professional development and ethical AI adoption.

The literature search was carried out using four major academic databases, including Scopus, Web of Science, IEEE Xplore, and Google Scholar, which are widely recognized for indexing high-quality peer-reviewed research in educational technology and artificial intelligence. The search strategy was based on carefully constructed Boolean keyword combinations to ensure comprehensive coverage of relevant studies. These included phrases such as “Artificial Intelligence in Education” AND “teacher competencies,” “AI ethics” AND “education” AND “responsible AI,” “AI literacy” AND “teacher training,” “generative AI” AND “education” AND “ethics,” and “AI integration” AND “classroom practices.”

The review focused on studies published between 2020 and 2025, a period characterized by rapid advancements in generative AI technologies and their increasing integration into educational systems worldwide. To maintain quality and relevance, only peer-reviewed journal articles and conference papers were included. Eligible studies were required to focus on AI in educational contexts, address ethical issues, teacher competencies, or AI literacy, and be published in the English language. Both empirical and conceptual studies were considered. Studies were excluded if they involved non-educational AI applications, were editorial or opinion-based, contained duplicate records, or lacked sufficient methodological clarity.

The initial search yielded 186 records. After removing duplicates and conducting title and abstract screening, 145 studies remained for further evaluation. A full-text assessment was then performed, resulting in 94 eligible articles. Following detailed eligibility screening, 52 studies were finally included in the qualitative synthesis. The PRISMA-based screening process ensured a transparent and systematic selection of literature, enabling the identification of key recurring themes across studies. The final synthesis revealed critical ethical concerns in AI-integrated education, including data privacy, fairness, accountability, transparency, inclusiveness, and human-centered AI design (Holmes et al., 2022; UNESCO, 2024; Nguyen et al., 2023).

For data analysis, thematic synthesis was employed following the framework proposed by Braun and Clarke (2006). A structured coding scheme was developed based on three core dimensions: AI ethical principles, teacher competency frameworks, and AI integration practices in educational environments. The coding process involved iterative comparison, refinement, and categorization of extracted themes to ensure conceptual consistency and analytical depth. The resulting themes were further validated through cross-comparison across studies, enabling the development of a robust conceptual foundation for the proposed ethical competency framework.

### 2.3 Development of the ethical competency framework

Based on the findings of the systematic literature review, a conceptual ethical competency framework was developed for teachers in Pakistan and Saudi Arabia. The framework was primarily developed using a deductive approach in which competency dimensions were derived and adapted from established international AI competency and responsible AI frameworks, including UNESCO’s AI Competency Framework for Teachers, Human-Centered AI principles, Responsible AI guidelines, and AI literacy literature (Holmes et al., 2022).

The framework was also conceptually informed by human-centered educational approaches and teacher competency theories, particularly those emphasizing ethical technology integration, digital literacy, and responsible pedagogical AI adoption. Existing literature suggests that effective AI integration in education requires not only technical AI knowledge but also ethical awareness, accountability, transparency, and inclusiveness (Pedro et al., 2019).

The proposed framework consists of two major dimensions: AI Integration Competencies and AI Ethical Competencies. These dimensions collectively address practical AI skills, AI-enhanced pedagogical design, ethical awareness, transparency, accountability, data privacy, equity, and inclusive AI-supported learning practices. The framework was specifically contextualized for the educational systems of Pakistan and Saudi Arabia to address local educational challenges, teacher preparedness limitations, and emerging AI adoption practices.

### 2.4 Pilot study procedure

A pilot study was conducted to examine the preliminary applicability, usability, and perceived ethical relevance of the proposed ethical AI competency framework for teachers in Pakistan and Saudi Arabia. The pilot was designed as an exploratory validation stage rather than a full-scale effectiveness study. Therefore, the findings are interpreted as preliminary evidence of teachers’ perceptions and experiences, not as conclusive evidence of long-term educational impact. The pilot involved 30 teachers from different educational levels and institutional contexts. Participants were selected through purposive sampling because the study required teachers who had basic familiarity with digital educational tools and were willing to engage in AI-supported teaching activities. This approach is appropriate for exploratory educational research where participants are selected based on their relevance to the phenomenon under investigation (Durgungoz and Kharrufa, 2026).

Over a two-week period, participants used selected AI tools for lesson planning, classroom activities, instructional support, assessment preparation, and professional development tasks. Before implementation, teachers were introduced to the ethical principles embedded in the proposed framework, including equity, inclusion, transparency, accountability, child safety, and data privacy. These principles are consistent with responsible AI and human-centered AI guidance in education (Cukurova and Miao, 2024; Holmes et al., 2022; Nguyen et al., 2023).

A mixed-methods evaluation was used to collect teachers’ feedback. Quantitative data were collected through a structured questionnaire measuring perceived effectiveness, usability, ethical compliance, satisfaction, and intention for future use. Qualitative data were collected through open-ended responses and semi-structured interviews to capture teachers’ experiences, challenges, ethical concerns, and suggestions for improvement. Quantitative data were analyzed using descriptive statistics in SPSS, while qualitative responses were analyzed through thematic analysis following the procedures of coding, theme identification, and interpretation (Braun and Clarke, 2006). This combination of survey and qualitative feedback enabled a more comprehensive understanding of how teachers perceived the framework during initial implementation.

### 2.5 Pilot study participants information

To evaluate the applicability and preliminary effectiveness of the proposed framework, a pilot study was conducted involving 30 teachers from different educational levels and institutional contexts in Pakistan and Saudi Arabia. The pilot study adopted a purposive sampling strategy, where participants were selected based on their familiarity with digital educational technologies and willingness to participate in AI-supported teaching activities. Purposive sampling is commonly used in exploratory educational research to obtain contextually relevant insights from participants with practical experience related to the study topic (Cohen, 1988).

**Figure 1:**
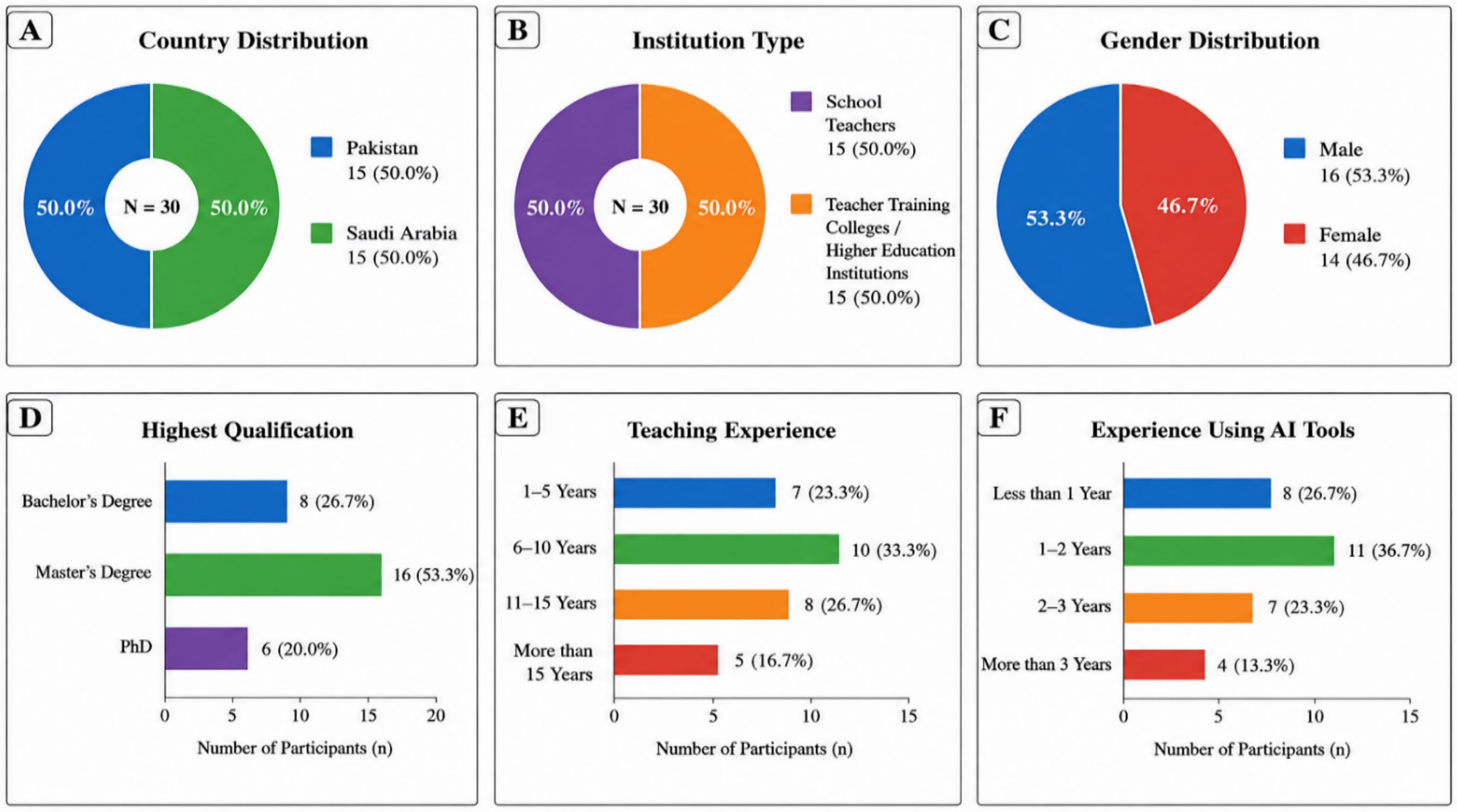
Demographic profile of participants.

The sample included teachers from schools, Teacher training college education institutions representing diverse academic disciplines and teaching experiences. Figure 1 presents the demographic characteristics of the participants involved in the pilot study. The sample consisted of 30 teachers, equally distributed between Pakistan (*n* = 15) and Saudi Arabia (*n* = 15). Participants were recruited from both schools (*n* = 15) and teacher training colleges/higher education institutions (*n* = 15) to ensure representation across different educational contexts. The sample included 16 male (53.3%) and 14 female (46.7%) teachers. Most participants possessed a Master’s degree (53.3%), followed by Bachelor’s degree holders (26.7%) and PhD holders (20.0%). Teaching experience varied across participants, with the largest group having between 6 and 10 years of experience (33.3%). All participants had attended at least one AI-related professional development activity and reported prior experience using AI tools in educational settings, making them suitable participants for evaluating the proposed ethical AI competency framework.

### 2.6 Survey tool and reliability

A structured survey instrument was developed to evaluate teachers’ perceptions of the proposed ethical AI competency framework. The questionnaire included five Likert-scale items rated from 1 = strongly disagree to 5 = strongly agree. The items measured perceived effectiveness, usability, adherence to ethical guidelines, overall satisfaction, and likelihood of future use. Example items included: “The AI tools enhance my teaching practice,” “The AI tools are easy to use in my classroom,” and “The AI tools support ethical and responsible teaching practices.” Open-ended questions were also included to collect qualitative feedback regarding implementation challenges, training needs, and ethical concerns. To strengthen content validity, the survey items were reviewed by experts in educational technology, AI ethics, and professional teacher development. Their feedback helped ensure that the questionnaire items were aligned with the study objectives and the proposed competency framework. Reliability was assessed using Cronbach’s alpha. The values for effectiveness, usability, and ethical compliance were 0.78, 0.81, and 0.84, respectively, indicating acceptable internal consistency for exploratory research (Hair et al., 2019). The test–retest reliability coefficient was 0.75, suggesting satisfactory response stability over time. The pilot study was not intended to generalize results to all teachers in Pakistan and Saudi Arabia. Instead, it provided preliminary evidence regarding the clarity, usability, and perceived ethical relevance of the proposed framework. This limitation is acknowledged to avoid overgeneralization and to support future large-scale validation studies.

### 2.7 Data analysis

The data analysis process in this study followed a mixed-methods analytical strategy integrating both quantitative and qualitative techniques to ensure methodological triangulation and comprehensive interpretation of results. Quantitative data collected through the structured questionnaire were analyzed using the Statistical Package for Social Sciences (SPSS, version 22). Descriptive statistical techniques, including frequencies, percentages, means, and standard deviations, were employed to evaluate teachers’ perceptions regarding the effectiveness, usability, ethical compliance, satisfaction, and future adoption of the proposed AI competency framework. Microsoft Excel was additionally used for preliminary data organization, data cleaning, tabulation, and graphical representation of demographic and response distributions prior to SPSS analysis, thereby ensuring data accuracy and improved visualization of trends.

For qualitative data analysis, responses obtained from semi-structured interviews and open-ended survey questions were analyzed using thematic analysis following the widely adopted framework of Braun and Clarke (2006). The analysis process included systematic coding, categorization of responses, and the identification of recurring patterns related to ethical concerns, teacher preparedness, implementation barriers, perceived usability of AI tools, and professional development needs. To enhance analytical rigor and consistency in theme development, initial coding and theme structuring were supported using ChatGPT as an assistive qualitative analysis tool. However, all generated themes were critically reviewed, validated, and refined manually by the researchers to ensure academic rigor, interpretative accuracy, and alignment with the study objectives.

In addition, ChatGPT was used as a supplementary tool to assist in the conceptual organization of qualitative insights and to support the drafting of visually structured representations of findings, including preliminary conceptual diagrams and figure layout suggestions. All visual outputs were further refined and reconstructed by the authors to ensure scholarly accuracy and compliance with academic publishing standards.

The integration of SPSS-based statistical analysis, Excel-based data management, and thematic qualitative synthesis enabled methodological triangulation, thereby strengthening the reliability, validity, and depth of the findings. This mixed-method integration allowed for a more holistic interpretation of teachers’ experiences and perceptions regarding AI integration in educational contexts. This analytical approach ensured that both numerical trends and qualitative insights were systematically integrated to provide a comprehensive understanding of the proposed ethical AI competency framework.

### 2.8 Ethical considerations

Ethical considerations were carefully maintained throughout the study. Participation was voluntary, and informed consent was obtained from all participants before data collection. Participants were informed about the objectives of the study, confidentiality of responses, and their right to withdraw from the study at any stage without penalty. No personally identifiable information was collected, and all responses were analyzed anonymously for research purposes only. Formal institutional ethical approval was not required because the study involved voluntary anonymous surveys with participants. However, we obtained prior permission from the Master Trainer and the Center in charge of the training institution for data collection during the teachers’ training program. The study adhered to responsible educational research practices and ethical principles associated with AI integration in education (Cukurova and Miao, 2024).

## 3 Proposed ethical competency framework

### 3.1 Thematic findings on ethical challenges of AI integration

The introduction of Artificial Intelligence (AI) in education is bringing meaningful new tools (among others) for both teachers and students. Artificial Intelligence (AI) can offer teachers plenty of opportunities like professional development support, instructional design improvements, personalization of student learning experiences, administrative decision-making assistance, and real-time feedback (Kim and Kwon, 2023). AI in education does bring these benefits but requires teachers to develop skills other than basic digital skills including data literacy, algorithmic knowledge, computational thinking and ethical AI usage (Kim and Kwon, 2023; Ng et al., 2023). Even with schools adopting AI tools, little emphasis were laid on preparing teachers and developing their AI competency (Edmett et al., 2023). A recent survey by UNESCO revealed that less than 10 per cent of educational institutions had formal policies on generative AIs. According to the literature, teachers must possess competency in handling AI applications, designing learning material supported by AI and performing a critical evaluation of activities enhanced by AI (Chiu, 2021; Mikeladze et al., 2024; Ng et al., 2023). In addition, teachers need to become experts in the ethical use of AI as part of a wider digital transformation effort (Kim and Kwon, 2023; Ng et al., 2023). For a successful integration of artificial intelligence, teacher readiness is critical. This is supported by the European Commission (2022) which calls for a proper understanding of the AI systems by the teacher and its safe application. AI technology helps reduce the pressure of teachers by taking over repetitive tasks and allowing them time for more imaginative and teaching jobs (Bryant et al., 2020). However, the fast development of AI, especially Large Language Models and similar tools, makes it difficult for educators to keep up (Bryant et al., 2020; Motlagh et al., 2023; Sandhu et al., 2024). To overcome these issues, a strong ethical competency framework is needed to sharpen the capacities of teachers in Pakistan and Saudi Arabia so they can effect an integration of AI in professional development and classroom practice in. The framework should take the needs of teachers and students into account, as teachers are said to be involved in the early phases of this integration.

The possibilities for education provided by AI are huge, but it also brings serious ethical issues with it. The usage of artificial intelligence in education grants students a personalized learning experience, enhances disability access, automates tasks, and more. However, it is not without concern. This includes fairy, privacy, teacher and transparency and teachers’ role. Artificial intelligence faces multiple challenges, like privacy problems due to large-scale collection and analysis of the data of students and teachers. This information can be misused and compromised without proper security. Alwakid et al. (2025) highlighted the use of emerging technologies such as blockchain is under consideration to secure data. Also identified within this are the need for a transparent data protection policy and consent mechanism. Bias in AI algorithms is another critical ethical issue. Poorly designed AI models create inequity in the learning experience. Vashishth et al. (2024) opine that while cryptographic algorithms can help address bias, developers should focus on fairness and inclusiveness. Ensure that AI-based learning platforms promote equity among all students, irrespective of their backgrounds. Automation of teaching tasks also poses challenges for the teachers. While AI can give criticism and streamline instructional plans, it’s possible for excessive use to hamper contact within the classroom. To protect and preserve the innovative ability of students, a suitable balance between the use of artificial intelligence and teacher involvement is required. AI should assist in teaching and learning process instead of replacing teacher. To address these ethical concerns, policymakers, teacher educators, and technology developers need to help articulate expectations and guidelines on privacy, bias, transparency, and professionalism. Pakistan and Saudi Arabia’s system of teacher education and training is also facing pedagogical problems in the use of AI (Kim et al., 2023; Motlagh et al., 2023). Pedagogical models should be based on ethical principles of truth, responsibility and the common good. Educators require training to utilize artificial intelligence in a way aligned with these values. For a successful execution one has to be technophisticated and thoroughly acquainted with enhances learning and ethics (Brossi et al., 2022; Mouta et al., 2024). Technical and systemic challenges are equally important. Leveraging AI in teacher education includes designing relevant systems, frameworks and interventions. There is a need for educator, government, and technology expert collaboration to ensure effective AI integration (Pedro et al., 2019). In Pakistan and Saudi Arabia, AI tools must also reflect the local educational context, teacher goals, and students’ learning goals. Curriculum design must include AI literacy for teachers and students (Hadi et al., 2023; Kim et al., 2023; Wang et al., 2024). AI concept and application have to be clear to the teachers. By preparing educators to use them responsibly, as well as equipping students for challenges of the future, developing AI competencies.

### 3.2 Comparative analysis of international AI ethics frameworks

As a result of the rapid growth of AI, countries and organizations are creating artificial intelligence ethics principles because of the multiple risks and safety concerns of AI. Many of the guidelines designed for responsible AI do consider fairness, transparency, accountability, etc. There are over 100 AI Ethics frameworks across the world. These frameworks get shaped by the social, legal, and cultural contexts of a particular region, industry, or sector. Key examples include the guidelines from the US, UK, China and other countries and sectoral guidance like healthcare, education and data privacy in guidelines from GE Healthcare, Google, and UNESCO. Pakistan and Saudi Arabia does not have its own set of AI ethics guidelines because at the moment there is not a comprehensive framework that meets the country’s cultural, educational and societal needs. Pakistan and Saudi Arabia must create frameworks for ethical AI use within education as AI technologies begin to integrate within teacher training and usage within the classroom. By establishing specific guidelines for the educational sector, Pakistan and Saudi Arabia can tackle the ethics of AI use for schools and teacher professional development in accordance with national values, educational priorities and cultural norms. This way, AI would bring benefits to classrooms without compromising on ethics and pedagogy (Younas, 2020). At present, Pakistan and Saudi Arabia uses guidelines established by big tech firms for adopting AI tools. Key stakeholders are recognizing the need for region-specific frameworks to address localized challenges in the education and technology mix. Their involvement has come to the fore through learning initiatives. Pakistan and Saudi Arabia possesses a distinctive character defined by population, possessions, perceived future, people, oppression, type, and influencer. It is the elemental essence of Pakistan and Saudi Arabia. Discussions that involve multiple stakeholders like government agencies, educational institutions and technology providers are being recognized as important for formulating policies for teacher training and classroom engagement. By developing such localized AI ethics principles for the educational field of Pakistan and Saudi Arabia, we will not only make the use of AI more responsible, but we will also make Pakistan and Saudi Arabia more digitally ready with a good repute. Teachers can use personalized ethical guidelines to integrate AI tools, protect students information, ensure justice, and promote transparency and accountability in education.

### 3.3 Synthesized ethical principles and competency dimensions

The ethical use of AI in education is provided by several international competency frameworks. As per the World Economic Forum, there are seven key principles guiding the implementation of AI in education. These are purpose, compliance, knowledge, balance, integrity, agency, and evaluation (Stefán, 2023). In the same vein, UNESCO’s AI in education competency framework calls for essential competencies development of educators for the effective use of AI tools. According to Nguyen et al. (2023), there are ethical principles for AI in education. In their paper, Nguyen et al. made a strong case for a global consensus on ethical guidelines. Moreover, these ethical guidelines can counter the ethical risks of AI technology [58]. Key principles include learner autonomy, transparency, data security and accountability. This aims to ensure that the AI tools are used for the benefit of all learners and the risks are minimized (Nguyen et al., 2023). UNESCO has also emphasized the need to look into these issues such as risks of privacy breach and bad implementations. This framework describes in detail the competencies that are relevant for integrating AI into teacher practices and classroom language education. Du Boulay (2022) examined how moral dilemmas regarding the use of AI in education have changed from the 1970s to the present. As per the research whose focus initially was learning tools for learners, is no longer the case now. Teachers and administrators’ tools have also assumed significance. Additionally, there has been an increased awareness of emotions and motivation. These changes though, raise concerns of autonomy, misuse of data, bias, and unfairness (du Boulay, 2022). The artificial intelligence systems require extensive student data (academic results, behavior, etc.) to work efficiently. This is a hurdle for them in employing the students. It is important for safeguarding trust and protecting the rights of the students (Nguyen et al., 2023). Studies show that measures such as anonymization, encryption, etc. are must for having reliable data protection. If the information that pops up in robots is biased, AI system cannot be unbiased. In essence, AI systems can suffer from bias due to a variety of sources, as discussed in the references (Mehrabi et al., 2021; Noble and Dubljević, 2022). It is a continual process to make things fair through checking and correcting.

**Figure 2:**
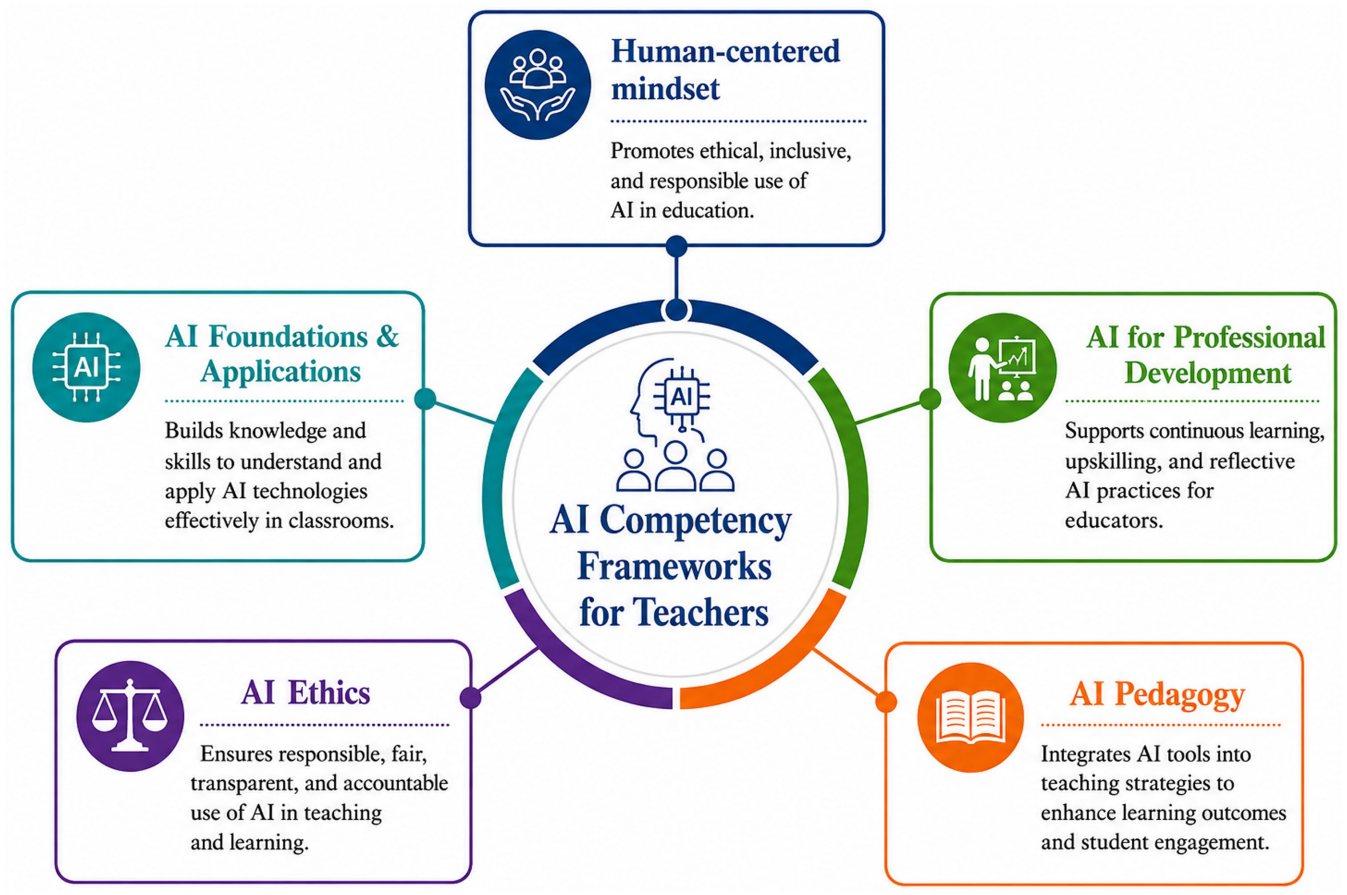
AI competency framework for teachers by UNESCO (2024).

Transparency is another essential priority, meaning AI-based decisions must be explainable. In turn, this will ensure trust and accountability. Nguyen et al. (2023) state that AI systems should be able to provide details on how the outputs are created or generated so that educators and learners understand how they happened. Especially in the case of failure of the AI System or generating unwanted results, it is equally important to assign accountabilities. The roles of developers, educators and students would be clarified for AI in these frameworks. Tang and Su (2024) made a systematic review of 32 publications to examine the ethical consequences and essential guidelines for AI in education. Researchers found five major ethical issues which include data privacy leak, algorithm bias, reduced autonomy, transparency deficit and academic fraud. Moreover, six ethical principles advised are fairness, transparency, privacy, autonomy, accountability, and beneficence. In addition, the study also indicated the need for follow up studies to understand ethics. Table 1 shows various competencies and their descriptions as depicted in Figure 2, sourced through the mentioned references. Studies by Adams et al. (2023) identified ethical principles for the introduction of AI in K-12 education. The principles include transparency, accountability, and prioritization of students’ interests. A similar investigation was carried out by Akgun and Greenhow (2022) concerning the ethical challenges associated with AI adoption in K-12 schools. Likewise, it was reported that bias, privacy, and transparency must be taken carefully.

**Table 1:** AI competencies summary.

Competency	Description	Source(s)
Equity and inclusion	Ensuring all students have equal access to language learning opportunities	(Nguyen et al., 2023; UNESCO, 2024)
Cultural sensitivity	Respecting and incorporating students’ cultural backgrounds in teaching methods	(Binns, 2018; Miao et al., 2021; Noble and Dubljević, 2022; UNESCO, 2024)
Transparency	Clearly communicating the objectives, methods, and outcomes of language programs.	(Floridi et al., 2018; Doshi-Velez and Kim, 2017)
Accountability	Holding educators and institutions responsible for ethical language teaching	(Mehrabi et al., 2021; Miao and Holmes, 2021; UNESCO, 2024)
Data privacy	Protecting students’ personal information and data collected during language learning	(Grizzle et al., 2014; Lipton, 2022)
Bias mitigation	Identifying and addressing biases in language teaching materials and methods	(Mehrabi et al., 2021; Noble and Dubljević, 2022)

**Figure 3:**
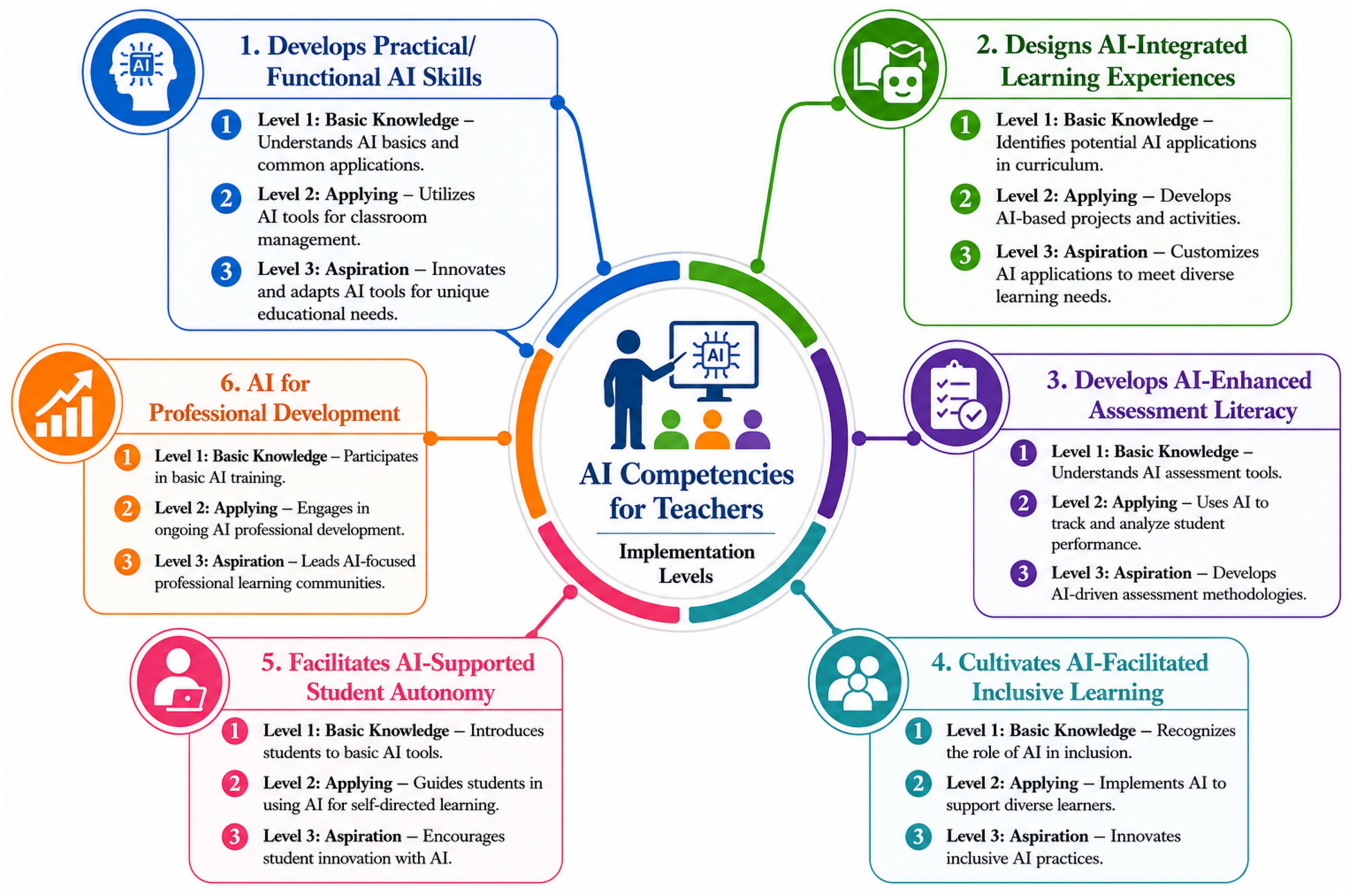
AI integration competencies and implementation levels for teachers.

Moreover, the UNESCO 2023–24 guideline outlines essential competencies for teachers and students to utilize AI in education ethically and responsibly, emphasizing human-centered approaches, AI foundations, ethics, and pedagogy see Figure 3.

As shown in Table 2, below are several framework and principles relevant to the use of AI in education to help Pakistan and Saudi Arabia teachers integrate AI. Artificial intelligence in education should be guided by ethics to ensure responsible use, privacy of student data and fair access to these technologies. Several important frameworks and laws provide guidelines and skills for educators and students in relation to ethics in the deployment of AI. According to the World Economic Forum’s first framework on AI in education, there are seven critical principles, they are; Purpose, Compliance, Knowledge, Balance, Integrity, Agency and Evaluation. The educational principles outlined here offer ethical guidance for realizing, refining and implementing AI that aligns with educational goals, compliance with policy as well as promoting AI literacy, balancing benefits and risks jeopardizing academic integrity, and ensuring human decision-making continuous impact assessment (Boza and Evgeniou, 2021; Díaz-Rodríguez et al., 2023; Stefán, 2023). This is to strengthen the privacy, bias and other counterproductive risks of AI. In addition, plus it maximizes the potential of AI. The UNESCO AI in UNESCO (2024) is a second framework that focuses on developing competencies of educators. The framework has different levels of competencies that will enable teachers to leverage AI tools for personalizing learning, automating administration and real-time feedback. It then emphasizes the vital ethical considerations that go into this process, including data privacy and algorithmic fairness, ensuring that AI is used to improve educational outcomes (Chiu et al., 2024; Díaz-Rodríguez et al., 2023).

**Table 2:** Key frameworks and principles for AI ethics in education.

Title	Type	Structure	Year	Geographic Scope	Education Sector	Developer	Target Group
Teaching AI for K-12	Principles and framework	Guidelines and competency levels	2023	International	K-12 education	TeachAI (ISTE and ASCD)	Educators and students
Ethical AI use in education	Principles	7 Key principles	2022	Global	General education	World economic forum	Educators, policymakers
UNESCO AI in education	Competency framework	Competency levels	2019	International	K-12 and higher education	UNESCO	Educators
Children’s internet protection act (CIPA)	Regulation	Safety and privacy requirements	2000	United States	K-12 education	U.S. Congress/FCC	Schools and libraries
Personal information protection law (China)	Law	Privacy and data protection rules	2021	China	General education	Government of China	Personal information handlers

The third framework TeachAI: Teaching AI for K-12 (2023), developed with ISTE and ASCD, offers useful advice on the use of AI for K-12 education. The project sequentially sets competency levels for the teachers and students, focusing on practical AI skill-sets, its ethical implications and the effective usage of AI tools in classrooms. This framework equipped teachers and students for future technological advancements as it advocates for AI literacy and recognizes the ethical challenges in AI (Grover, 2024). The fourth framework is the Children’s Internet Protection Act (CIPA), which was enacted by the United States in 2000. In simple terms, CIPA sets the safety and privacy conditions that apply to K–12 schools and libraries. This framework is important for establishing child safety on technologies used by the schools as well as the libraries. Further, it emphasizes monitoring harmful and obscene content (Jaeger and McClure, 2004). In comparison to ADE, China’s Personal Information Protection Law (Calzada, 2022) is more stringent. It has outlawed any data collection and processing which may adversely impact the rights and interests of individuals in education area. As such, educational institutions must implement data protection laws when making use of AI technologies (Calzada, 2022; Feng, 2019). Advanced countries such as the USA, Finland, China, Singapore place emphasis on developing AI literacy in education along with ethical competencies (Pedro et al., 2019; Rigley et al., 2024; Schiff, 2022). United States Department of Education AI is about equipping educators and learners with skills to harness AI and other technologies for the future (Murphy, 2019). In its national AI strategy, Finland focuses on developing AI skills for teachers and students at an early age (Mertala et al., 2022). Likewise, AI education in the national agenda to develop the AI competency of teachers and students is at the core of China’s policies (Knox, 2020; Yang, 2019). According to Table 2 various frameworks show support for ethical competencies for AI integration in education. They will guide a teacher in Pakistan and Saudi Arabia to use AI responsibly, ethically, and effectively.

### 3.4 Proposed ethical competency framework

**Figure 4:**
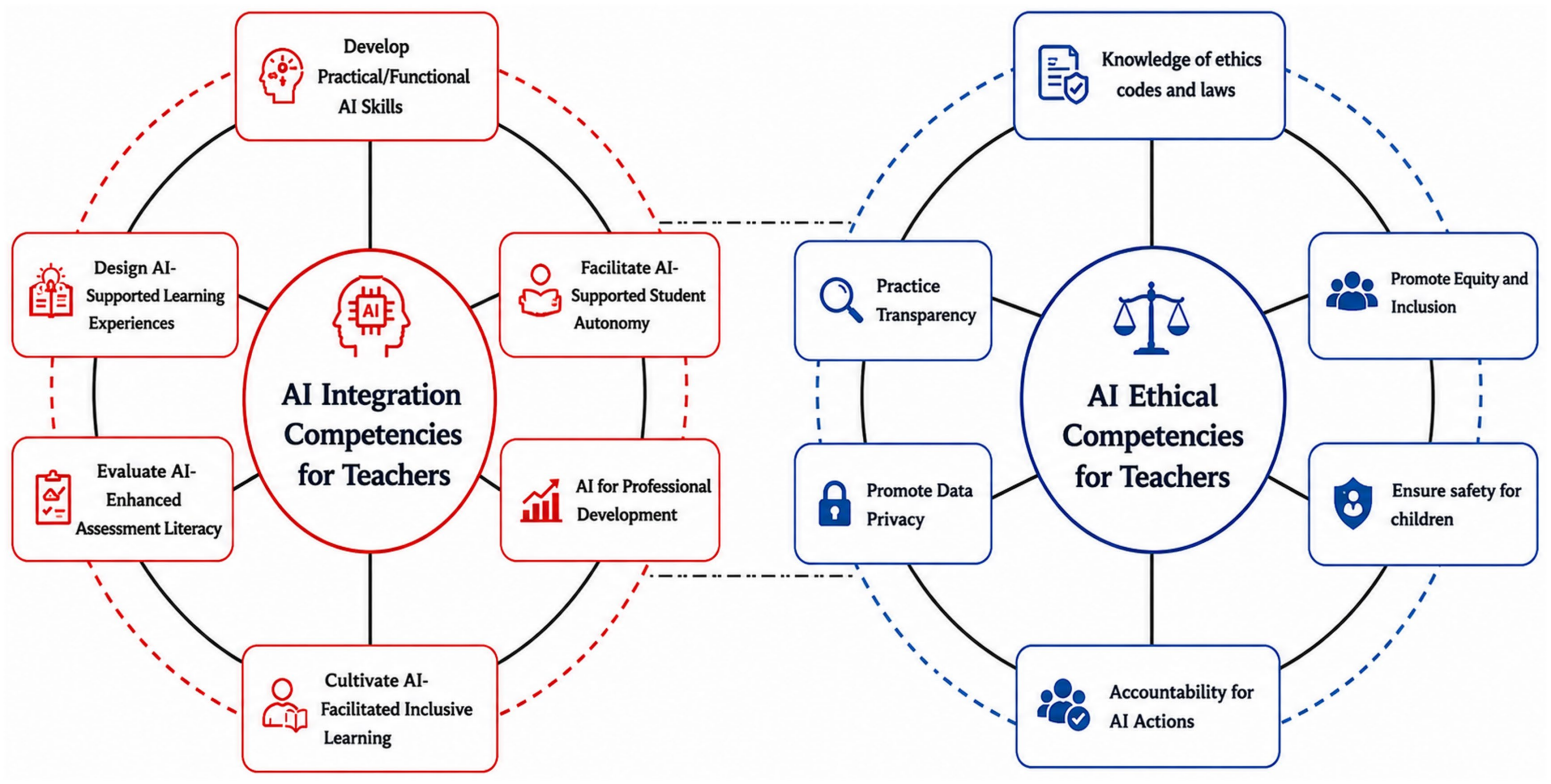
Proposed ethical competency framework for AI integration for Pakistan and Saudi Arabia’s teachers.

Based on an extensive review of existing literature, the ethical competency framework was designed for the use of AI in education as shown in Figure 4. We looked at competency frameworks and guidelines from UNESCO and research studies in the fields of education and artificial intelligence. A literature review enabled the identification of essential ethical competences of use of AI in education emphasizing different global frameworks and initiatives. Through examining studies and guidelines from various international organizations and researchers, the authors (Nguyen et al., 2023) identified the key ethical competencies for AI in education. This framework has been developed for the Pakistan and Saudi Arabia context, considering the local education requirements, teacher readiness and challenges of AI in the classroom. The purpose of this document is to provide practical advice to teachers on the responsible and effective use of AI tools. A framework was built to inform teachers in Pakistan and Saudi Arabia to use AI in education ethically and responsibly. The framework of conceptual ethical competency will help teachers in Pakistan and Saudi Arabia. This framework acknowledges educational local realities, helping to enact AI technologies in a way that makes teaching and learning happen. In the segments that follow, each competency along with its description appears, directing the way for teachers to develop an ethical awareness, digital literacy, and capabilities to work in an AI-enabled classroom.

As artificial intelligence (AI) is increasingly used in education around the world, it is crucial that competencies are developed to help teachers use these technologies effectively and ethically. In terms of education in Pakistan and Saudi Arabia, these competencies are international in nature and applicable in the local educational environment. Educators who want to use AI to facilitate teaching and learning must have the following competencies. As summarized in Table 3 and Figure 3, each competency is presented below and implemented in three progressive phases.

**Table 3:** AI integration competencies (summary).

Competency	Objective	Implementation	Rationale
Develops practical/functional AI skills	Equip teachers with basic AI skills for classroom application.	Level 1: Basic Knowledge – Understands AI basics and common applications. Level 2: Applying – Utilizes AI tools for classroom management. Level 3: Aspiration – Innovates and adapts AI tools for unique educational needs.	Basic AI literacy enables teachers to effectively incorporate AI tools, enhancing their teaching methods and improving student engagement (UNESCO, 2021).
Designs AI-integrated learning experiences	Create engaging and interactive learning environments using AI.	Level 1: Basic Knowledge – Identifies potential AI applications in curriculum. Level 2: Applying – Develops AI-based projects and activities. Level 3: Aspiration – Customizes AI applications to meet diverse learning needs.	AI can personalize learning experiences, making education more engaging and effective for students of different learning styles (Akgun and Greenhow, 2022).
Develops AI-enhanced assessment literacy	Improve assessment accuracy and feedback using AI tools.	Level 1: Basic Knowledge – Understands AI assessment tools. Level 2: Applying – Uses AI to track and analyze student performance. Level 3: Aspiration – Develops AI-driven assessment methodologies.	AI helps in providing real-time feedback and detailed analysis, facilitating better understanding of student progress (UNESCO, 2021).
Cultivates AI-facilitated inclusive learning	Ensure all students benefit equally from AI advancements.	Level 1: Basic Knowledge – Recognizes the role of AI in inclusion. Level 2: Applying – Implements AI to support diverse learners. Level 3: Aspiration – Innovates inclusive AI practices.	AI can support differentiated instruction, making learning accessible to students with varying needs and abilities (Akgun and Greenhow, 2022).
Facilitates AI-supported student autonomy	Empower students to use AI tools independently.	Level 1: Basic Knowledge – Introduces students to basic AI tools. Level 2: Applying – Guides students in using AI for self-directed learning. Level 3: Aspiration – Encourages student innovation with AI.	Encouraging autonomy in AI use prepares students for a future where AI is integral to many aspects of life and work (UNESCO, 2021).
AI for professional development	Continuously improve AI-related skills and knowledge.	Level 1: Basic Knowledge – Participates in basic AI training. Level 2: Applying – Engages in ongoing AI professional development. Level 3: Aspiration – Leads AI-focused professional learning communities.	Ongoing professional development ensures that teachers stay current with AI advancements and best practices in education (UNESCO, 2021).

#### 3.4.1 Develops practical/functional AI skills

The first competency features the development of practical and functional AI skills so teachers can use it effectively in the classroom. A competency that requires acquisition of technical skills to be able to operate AI tools and applications that enhance teaching and learning. Teachers in Pakistan and Saudi Arabia should learn these practical skills to implement AI tools in a classroom setting. Teachers must understand how the AI algorithms work and become adept at using an AI-based educational application. When teachers build useful AI skills, they can better improve their teaching strategies and offer personalized learning experiences to students (Dahri et al., 2024b; Al-Rahmi, 2026c).

We propose a three-tiered approach for this competency:

1. In the Basic Knowledge Stage, teachers will learn about the AI concepts that are relevant to the education sector. They will become familiar with the relevant terminology along with understanding its role in education and learning.
2. Teachers start to engage artificial intelligence tools to help them manage their class facilities which include grading, attendance, lesson organizing and added instruction.
3. At this point, educators show their innovative potential through adaptation of AI tools to meet specific and contextualized educational needs.

Ensuring AI skills for teachers will help them use AI tools better and thus improve teaching and increase student engagement (UNESCO, 2021). Previous literature indicates that educators should have adequate practical skills of AI to make the best use of technology (Dahri et al., 2024c). In Pakistan and Saudi Arabia, where access to the latest technology may be limited, the importance of this skill set can help to maximize available artificial intelligence to create interactive, personalized, and ethically responsible learning experiences for students (Alwakid et al., 2025; Dahri et al., 2025).

#### 3.4.2 Designs AI-integrated learning experiences

The second competency focuses on developing lesson plans and learning activities that use AI tools in a way that has no ethical concerns and enhances teaching and learning in the Pakistan and Saudi Arabia classroom. Teachers must be equipped to design learning experiences using AI to facilitate the engagement of the students and the enhancement of learning outcomes. AI-driven platforms can create customized educational content based on individual student needs. According to Park et al. (2023), adaptive learning platforms can offer personalized and responsive learning experiences (Park et al., 2023). In Pakistan and Saudi Arabia, lesson planning using AI can be helpful in meeting the diverse requirements of students, promoting inclusiveness and enhancing quality education (Mukhamadiyeva and Hernández-Torrano, 2024).

For this competency, we propose a three-tiered approach:

1. The first stage is Basic Knowledge/Understanding where teachers identify AI applications that can be ethically used in their curriculum.
2. In the application stage, you create your own AI-based projects and classroom activities that promote interactive, personalized, and ethical learning.
3. Teachers customize AI applications to meet the needs of their students. Each student is different and has a different learning style, language background and ability. A student must not be left behind, this ensures that no student is left behind, while being ethical.

The integration of AI in education can personalize learning experiences, helping the teaching to be more engaging, effective, and inclusive (Akgun and Greenhow, 2022). According to some research, AI-enhanced technology can help students learn better and faster through adaptive feedback and interactive tools. Educationalists in Pakistan and Saudi Arabia believe that the use of AI-integrated learning experiences can help enhance equitable access, enhance learner engagement and improve educational outcomes in a classroom where students come from different languages and socio-economic backgrounds.

#### 3.4.3 Develops AI-enhanced assessment literacy

The 3rd competency is understanding how to ethically use AI tools to assess students’ performance and learning levels in Pakistan and Saudi Arabia. When teachers develop assessment literacy to capitalize on artificial intelligence (AI) technologies, they can create, deliver and analyze assessments more accurately, quickly and personally. Instructors can get automated scoring and feedback as well as data-driven insights with AI tools that help them. According to Parsa et al. (2021), AI-powered assessment tools have a lot of advantages including delivering objective assessments (Parsa et al., 2021). When it comes to Pakistan and Saudi Arabia, helping functionalities of AI for developing assessment literacy in teachers can make accurate and efficient assessments of students while ensuring warrantable use of student data. Teachers who develop this competency use AI tools to enhance assessment practices in a responsible manner.

This competency can be structured into three levels:

1. Teachers know what they are capable of and what it means to use this technology ethically.
2. Teachers use AI technology at the application level to monitor and analyze how students perform. This helps them make decisions about a student’s learning journey, while keeping data safe from privacy leaks.
3. The aim of teachers is to create assessment techniques that are AI-based and which can help teachers assess the learning outcomes of students effectively and ethically. The assessment must target All learners should be provided with timely feedback, personalized learning pathways and actionable insights.

It helps enhance the feedback loop, improve the understanding of learner progress, and renew assessment. In Pakistan and Saudi Arabia, assessment literacy enhanced through artificial intelligence will enable a richer understanding of student learning by providing the means for educational decisions based on data (Hooda et al., 2022; Parsa et al., 2021; Shin et al., 2022; Swiecki et al., 2022).

#### 3.4.4 Cultivates AI-facilitated inclusive learning

Competency four states the involvement of AI tools in attribution to all students with learning differences in an ethical and responsible manner. AI is capable of creating a culture of inclusive education through the provision of technologies. Teachers have to develop the competency to use AI tools to promote equity so every student can benefit from personalized and accessible learning (El Naggar et al., 2024; Meng et al., 2022). AI-powered tools like speech-to-text and text-to-speech apps can help students with disabilities or language problems. According to studies, we can use AI to build inclusive educational settings, eliminating barriers to learning (Kazimzade et al., 2019; Knox et al., 2019; Sharma, n.d.; Xia et al., 2022). In Pakistan and Saudi Arabia, the AI technology adoption can help promote inclusiveness in learning and access to equal level of education for EVERYONE. This competency is structured into three levels:

1. At a basic knowledge level teachers are aware of the potential for AI to boost inclusiveness and understand how AI can help learners with diverse needs.
2. At the application level, teachers use AI tools to help students with learning disabilities receive equal opportunities in learning.
3. Teachers devise new ways of using AI to provide customized educational experiences for differently able and children in need of additional support.

AI technology allows teachers to differentiate instruction in classrooms so that all students have an accessible, engaging and supportive space. In Pakistan and Saudi Arabia, the ethical use of AI for inclusivity of resources can help mitigate the gaps and help achieve equitable language learning outcomes.

#### 3.4.5 Facilitates AI-supported student autonomy

The fifth competency highlights utilizing AI tools to promote ethical and responsible independent learning and self-regulation in students. Teachers must help students take control of their learning. This can be done easily with AI tools that help them to personalize their learning. Research by Alshumaimeri and Alshememry (2023) and Feng (2023) shows that AI can support the independence of students by allowing them to engage in autonomous activities (Alshumaimeri and Alshememry, 2023; Feng, 2023). In Pakistan and Saudi Arabia, AI can enhance learner-centered education through fostering student autonomy. This competency involves helping students use AI responsibly and ethically to support their learning. The three levels of this competency are:

1. The basic knowledge level for teachers means they will be exposed to AI tools and be able to use them to learn.
2. At the application level, teachers encourage students to utilize AI for self-learning. This enhances the students’ self-learning while also evoking a sense of individuality and accountability.
3. In the aspiration level, the teachers always asked students to perform innovation with AI and use these tools to learn new things and solve problems. Furthermore, it encouraged students to create their learning opportunities independently.

Encouraging students’ autonomy supported by AI prepares them for future professions through critical thinking, plan including identify information, solve problem and organize information skills. Moreover, these skills will help students to adapt their self-regulation and behavior (UNESCO, 2024; Robert, 2024). This competency in the Pakistan and Saudi Arabia system ensures that students will take control of their own learning through the use of AI-based technology to improve their language skills and learning.

#### 3.4.6 AI for professional development

According to the sixth competency, teachers should use AI tools for their continuous learning and professional development so that they are ethically and effectively prepared for AI usage in the classroom. There is need of continual professional development through which teachers must stay updated with new AI tools, techniques and their applications. Opportunities for ongoing education, including workshops, virtual courses, and AI-based training systems, can assist teachers in improving their abilities and responsibly utilizing AI. Professional development of teachers is essential for the effective use of AI in their academic curriculum. In Pakistan and Saudi Arabia, if proper professional development programs on AI are introduced, the teachers may represent it ethically. This competency is structured into three levels:

1. Trainers attend basic training on AI concepts and tools for education and their ethical use of it.
2. As AI continues to evolve, it is the responsibility of every teacher to be aware of AI developments and learn how to use them in the classroom to enhance student learning in an ethical manner.
3. Teachers take leadership roles to guide community of practice professional learning in ethical use of artificial intelligence, mentoring and sharing best practices with peers.

Continuing professional development ensures that teachers maintain awareness of emerging AI trends and understand the ethics and best practices for the use of AI in the classroom (Dahri et al., 2023). In Pakistan and Saudi Arabia, where opportunities for professional development may be limited, AI-based solutions can offer personalized, scalable training that enables teachers to continuously improve their skills and knowledge and use AI ethically in education.

To use artificial intelligence technologies appropriately and efficiently in classrooms, users need a certain combination of competencies that include ethical and practical ones. In Pakistan and Saudi Arabia, it is essential that teachers possess these competencies to ensure that AI-based teaching and learning is in line with local needs. With the help of these skills, teachers can embrace AI to make education more inclusive and personalized. It will also help students and teachers achieve their tasks with more ease and efficiency.

### 3.5 Ethical competency dimensions

**Figure 5:**
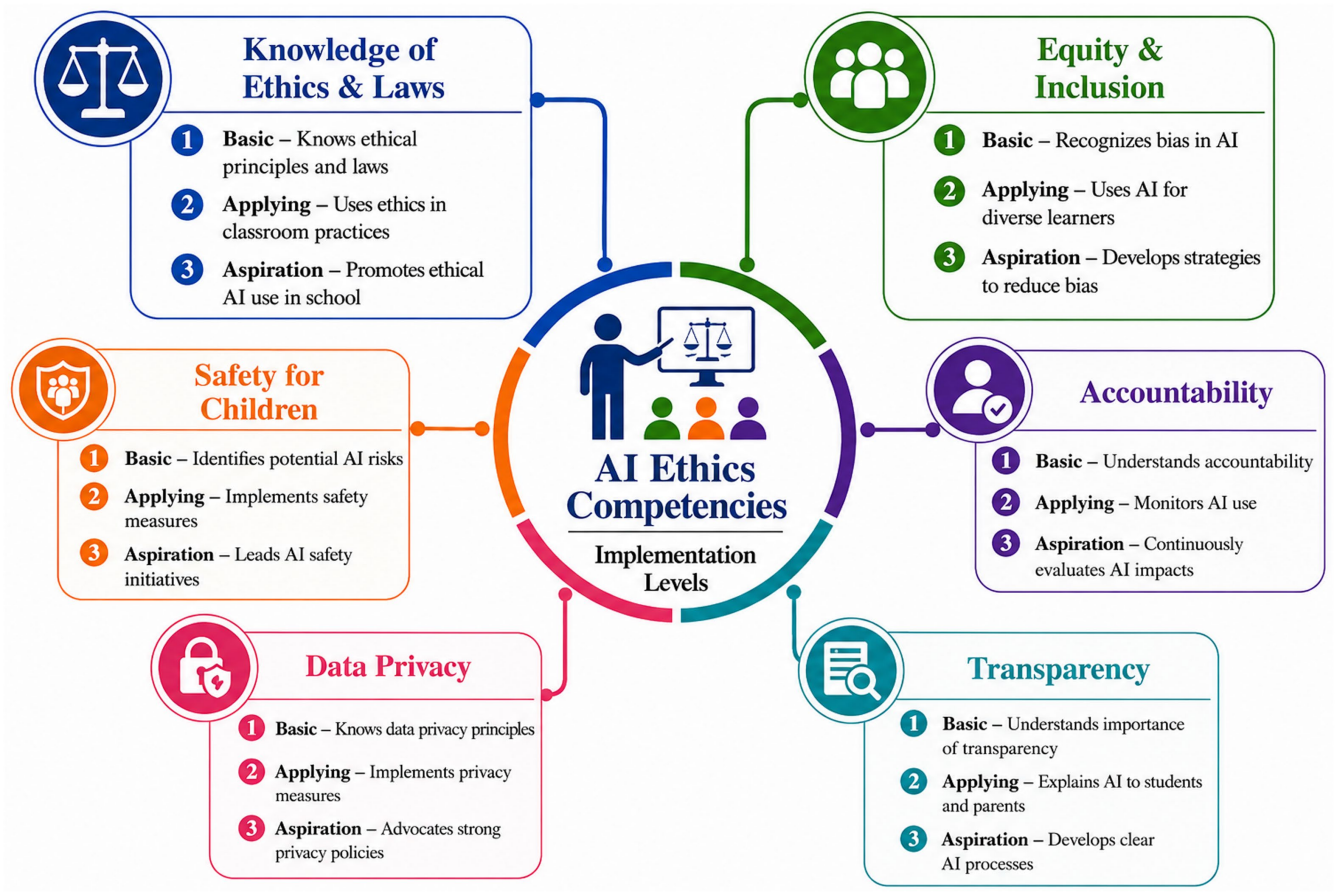
Proposed AI ethics competencies framework and implementation levels for teachers.

The use of AI in education surfacing ethical issues associated with the technology. To handle this responsibly, teachers in Pakistan and Saudi Arabia need to develop particular ethical competencies which meet global standards but yet satisfy local educational needs. The teaching profession must be informed by specifications by UNESCO, European Commission and others. These are the essential competences for the responsible use of artificial intelligence in teaching. The implementation of each competency has tri-phased (knowledge, abilities and attitude), which is shown in the Table 4 and Figure 5.

**Table 4:** AI ethics competencies (summary).

Competency	Objective	Implementation Levels	Rationale
Knowledge of ethics and laws	Understand ethical guidelines and legal rules for AI use	Level 1: Basic – Knows ethical principles and laws. Level 2: Applying – Uses ethics in classroom practices. Level 3: Aspiration – Promotes ethical AI use in school	Helps teachers use AI responsibly and fosters a culture of ethical use (UNESCO, 2021)
Equity and inclusion	Ensure AI supports all students fairly	Level 1: Basic – Recognizes bias in AI. Level 2: Applying – Uses AI for diverse learners. Level 3: Aspiration – Develops strategies to reduce bias	Prevents AI from reinforcing inequalities and supports diverse learning needs (European Commission, 2022)
Safety for children	Protect students from AI-related risks	Level 1: Basic – Identifies potential AI risks. Level 2: Applying – Implements safety measures. Level 3: Aspiration – Leads AI safety initiatives	Ensures students are safe from harmful content and misuse of AI (Akgun and Greenhow, 2022)
Accountability	Ensure AI is used responsibly	Level 1: Basic – Understands accountability. Level 2: Applying – Monitors AI use. Level 3: Aspiration – Continuously evaluates AI tools	Guarantees AI benefits students and is used ethically (UNESCO, 2021)
Data privacy	Protect student data and comply with privacy laws	Level 1: Basic – Knows data privacy principles. Level 2: Applying – Implements privacy measures. Level 3: Aspiration – Advocates strong privacy policies	Maintains trust and meets legal requirements (European Commission, 2022)
Transparency	Make AI processes clear and understandable	Level 1: Basic – Understands importance of transparency. Level 2: Applying – Explains AI to students and parents. Level 3: Aspiration – Develops clear AI policies	Builds trust and understanding of AI decisions (UNESCO, 2021)

#### 3.5.1 Knowledge of codes of ethics and laws

Understanding the ethical codes, laws, and regulations developing in a restrictive manner and implementing AI systems in education is the first ethical competency (Ghoz and Hendawy, 2023). Teachers understand and comply with the legal framework to prevent misuse and protect the rights of students in the institution. Understanding such codes and laws helps build an integrity culture at the place of education (Huriye, 2023). At the basic level of knowledge, teachers are familiar with basic ethical rules and laws that apply to the situation, including data privacy, consent and the ethics of AI decision-making. At the applying stage, teachers use these ethical guidelines in the classroom. The idea is to ensure that the AI tools are used ethically. Teachers’ Profession: At the aspiration level, teachers promote the ethical use of AI in their school communities, encouraging best practice and influencing school policy. Teachers who understand and apply ethical and legal principles in AI usage can use AI responsibly and ethically while promoting the moral and ethical use of AI in education (UNESCO, 2024).

#### 3.5.2 Ensures equity and inclusion

The focus of the second ethical competency is to develop equity and inclusiveness through AI in classrooms. Teachers should be aware of the biases present in AI and take whatever measures are necessary to ensure equal access for students (Pedro et al., 2019). It is important to develop this competency in order to prevent or lessen educational inequalities using AI (Huriye, 2023). At the basic knowledge level, teachers are aware that there is a chance of bias in AI systems, and that at some level, it is important to address this. During the applying stage, teachers are using AI tools to help different learners as they adapt strategies to different learners. At the aspiration level, teachers create and advocate for strategies to reduce AI bias so AI can help drive equitable results and not reinforce inequalities. According to sources, ensuring equity and inclusion by AI technology prevents discrimination, helps diverse learning needs and fosters a more inclusive one. UNESCO also emphasizes use of AI to boost inclusivity and equitable learning outcomes. In Pakistan and Saudi Arabia’s multicultural and multilingual setting, the employability of AI to fulfill the diverse need of students is a must.

#### 3.5.3 Ensures safety for children

The third competency is to make sure that children are safe when they are using AI at schools (Miao and Holmes, 2021). This entails working to understand and lessen the risks from AI exposure to, for example, inappropriate and harmful content, privacy and data security issues. AI tools should be used only in the order of 8 students’ safety and well-being (Ghoz and Hendawy, 2023). Teachers can be exposed to harmful content on the Internet. Their data can be leaked or hacked. Algorithms used to function or operate the Internet are biased. At the applying stage, teachers take safety measures such as adding filters to block content, monitoring the AI and students, and safely processing and storing the students’ data. The teachers take initiatives to enhance the AI safety of their school. It also priorities rules that protect the students. Effective safety measures ensure security in learning and prevent the misuse of AI (Akgun and Greenhow, 2022). According to a UNESCO report (2021), students should be protected from the harms of artificial intelligence. A report by the European Commission (2022) recommended the implementation of strict safety and monitoring measures. In Pakistan and Saudi Arabia, where digital skills and infrastructure are still in development, the need for safe AI is crucial in order to protect the student body and enhance their learning experience.

#### 3.5.4 Accountability for children

The fourth ethical competency is accountability of teacher as per the AI tools they use and their impact on the learner. This means that the AI used must be transparent and fair and minimize possible harms (Schiff, 2022). To a basic knowledge, teachers have a perception that there is accountability in using AI but monitoring and evaluation of AI. During the applying stage, teachers check AI tools to make sure they do not violate ethical standards. At this level, teachers are constantly evaluating and improving their AI practices to ensure that they are responsible. Accountability ensures that AI tools to be used properly for educational purposes and benefits students (UNESCO, 2021). UNESCO notes that AI applications should be transparent and accountable (UNESCO, 2024), while the European Commission demands specific insights into harm caused (Miao and Holmes, 2021). In Pakistan and Saudi Arabia, it is important to inspire trust in AI and education by a culture of accountability and transparency.

#### 3.5.5 Protects data privacy

According to Bećirović (2023), the fifth ethical competency states that teachers must ensure students’ data privacy. This competency requires familiarity with the laws regulating the data of students to secure data and make it safe for use in education (Torres-Hernández and Gallego-Arrufat, 2022). According to UNESCO, a strong data protection regime prevents breaches of privacy and ensures responsible use of data. The European Commission does overreporting and stresses that it expects full conformity with data protection and other laws and good practice (Irfan and Murray, 2023). In Pakistan and Saudi Arabia, where regulations related to data privacy have yet to be confirmed, student data privacy is vital. Teachers understand the fundamental principles related to privacy, which include particular laws and ethics, at the basic knowledge level. At the applying stage, the teachers take measures to maintain privacy hosting data securely, taking student permission before using any data, and anonymizing data. At the aspiration level, teachers advocate for comprehensive data privacy policies, while ensuring the highest standards for student data. The protection of data privacy builds trust and responsible use of AI (Miao and Holmes, 2021; Miao et al., 2021).

#### 3.5.6 Practices transparency

The sixth ethical competency related to AI concerned transparency in their use. This means the communication of how the AI tool works and why it decides the way it does should be explicit (Khosravi et al., 2022). All teachers must make the parents, students, and stakeholders understand the role of AI in the classroom so that they trust it (Holmes et al., 2022). At beginner knowledge, educators see the need for transparency in algorithm use by AI. At the applying stage, they communicate the ai process to the students as well as the parents. The schools explained how these tools work and how they will help the students learn with ease. At the aspiration level, teachers will develop clear policies and practices around the use of AI. When university lecturers can trust AI systems, they can, in turn, teach students how to engage positively with them (Vössing et al., 2022). UNESCO indicates the importance of transparency in the artificial intelligence (AI) system for making it ethical (Miao and Holmes, 2021). To build confidence among students, parents and educators in Pakistan and Saudi Arabia, transparency in the education system is important.

AI is becoming an important part of education. But we need a structural framework of ethical competencies for using AI as education tool. In Pakistan and Saudi Arabia, these are the teacher competencies: knowledge of codes and laws of ethics, ensuring equity and inclusion, ensuring safety for children, accountability for children, protecting data privacy and practicing transparency. In addition to meeting international standards, they are also adapted to local educational and cultural environments, which provides educators with a framework for working ethically with AI. Teachers can make sure that AI tools improve learning results, protect students’ rights, guarantee fairness, and keep faith in educational technology by developing these features.

## 4 Results

### 4.1 Quantitative findings

**Figure 6:**
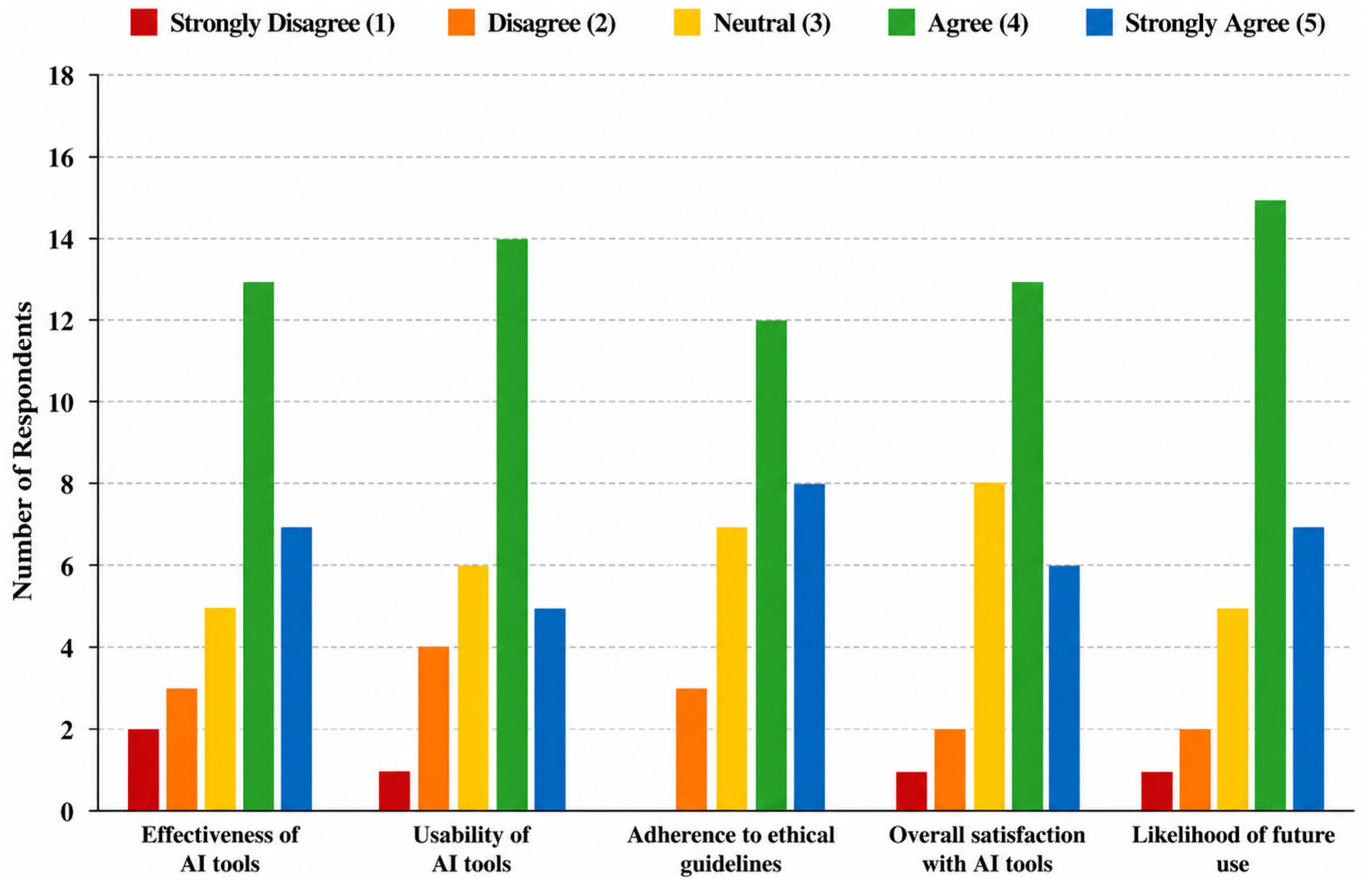
Quantitative findings.

Table 5 presents the quantitative findings obtained from the 30 participating teachers involved in the pilot implementation study conducted in Pakistan and Saudi Arabia. The findings indicate moderately positive perceptions regarding the usability, effectiveness, and ethical applicability of AI-supported educational practices within the proposed competency framework as presented in Figure 6.

**Table 5:** Quantitative survey results.

Metric	Strongly disagree (1)	Disagree (2)	Neutral (3)	Agree (4)	Strongly agree (5)	Mean	SD
Effectiveness of AI tools	2	3	5	13	7	3.73	1.09
Usability of AI tools	1	4	6	14	5	3.63	0.99
Adherence to ethical guidelines	0	3	7	12	8	3.83	0.96
Overall satisfaction with AI tools	1	2	8	13	6	3.70	0.97
Likelihood of future use	1	2	5	15	7	3.83	0.97

The mean scores for effectiveness (M = 3.73, SD = 1.09), usability (M = 3.63, SD = 0.99), ethical compliance (M = 3.83, SD = 0.96), overall satisfaction (M = 3.70, SD = 0.97), and likelihood of future use (M = 3.83, SD = 0.97) suggest that most participants viewed the proposed framework positively. In particular, ethical compliance and future intention to use AI tools demonstrated the highest mean scores, indicating that participants perceived the framework as supportive of responsible and ethically informed AI integration practices.

Approximately 60% of participants agreed or strongly agreed that AI tools improved teaching practices and classroom engagement. Similarly, nearly 63% of respondents perceived the AI tools as usable and supportive within their teaching activities. Furthermore, approximately 67% of teachers agreed that the AI-supported activities aligned with ethical principles related to transparency, fairness, accountability, and responsible AI usage.

The standard deviation values ranging from 0.96 to 1.09 indicate moderate variability in participants’ responses, suggesting that although most teachers demonstrated positive perceptions, some participants remained uncertain or cautious regarding AI implementation. This variation may be associated with differences in prior AI experience, institutional readiness, and digital competency levels among teachers.

The quantitative findings provide preliminary evidence that teachers perceived the proposed framework as useful, ethically relevant, and potentially supportive for AI integration in educational settings. However, given the exploratory nature of the pilot study and the relatively small sample size, the findings should be interpreted cautiously and should not be generalized beyond the current study context. Future large-scale studies involving more diverse educational institutions are recommended to further validate the framework and examine its long-term educational impact (Creswell and Clark, 2017; Hair et al., 2019).

### 4.2 Qualitative findings

Qualitative feedback obtained through semi-structured interviews and open-ended survey responses provided deeper insights into teachers’ experiences regarding the implementation of AI tools within the proposed ethical competency framework. The qualitative findings were analyzed using thematic analysis following the procedures suggested by Braun and Clarke (2006), where responses were coded, categorized, and grouped into recurring themes. Several major themes emerged from the analysis, including perceived instructional benefits, ethical awareness, implementation challenges, and the need for continuous professional development.

One of the most frequently identified themes was the perceived improvement in teaching and learning practices through AI-supported educational activities. Many teachers reported that AI tools assisted them in lesson planning, personalized learning support, automated feedback, and classroom engagement. Participants indicated that AI technologies helped reduce repetitive instructional tasks and enabled more adaptive teaching approaches aligned with individual student needs. These findings are consistent with previous studies highlighting the role of AI in enhancing personalization, learner engagement, and instructional efficiency in educational settings (Holmes et al., 2022; Chiu et al., 2024).

Another important theme involved ethical awareness and responsible AI usage. Teachers generally perceived the proposed framework as useful for guiding ethical AI integration practices, particularly regarding transparency, fairness, accountability, inclusiveness, and data privacy. Several participants emphasized that the framework increased their awareness of responsible AI practices and encouraged more cautious and reflective use of AI technologies in classroom activities. These findings align with UNESCO’s recommendations emphasizing the importance of ethical AI competencies for educators (Cukurova and Miao, 2024; Nguyen et al., 2023).

Despite the positive perceptions, participants also identified several implementation challenges. One recurring concern was limited prior knowledge and practical experience with AI technologies. Some teachers reported difficulties in understanding AI tools, evaluating AI-generated outputs, and applying ethical principles consistently during instructional activities. Participants also expressed concerns regarding institutional readiness, technical support limitations, and unequal access to digital infrastructure. Similar barriers have been reported in previous AI-in-education studies, particularly within developing educational environments undergoing digital transformation (Pedro et al., 2019; Motlagh et al., 2023).

**Figure 7:**
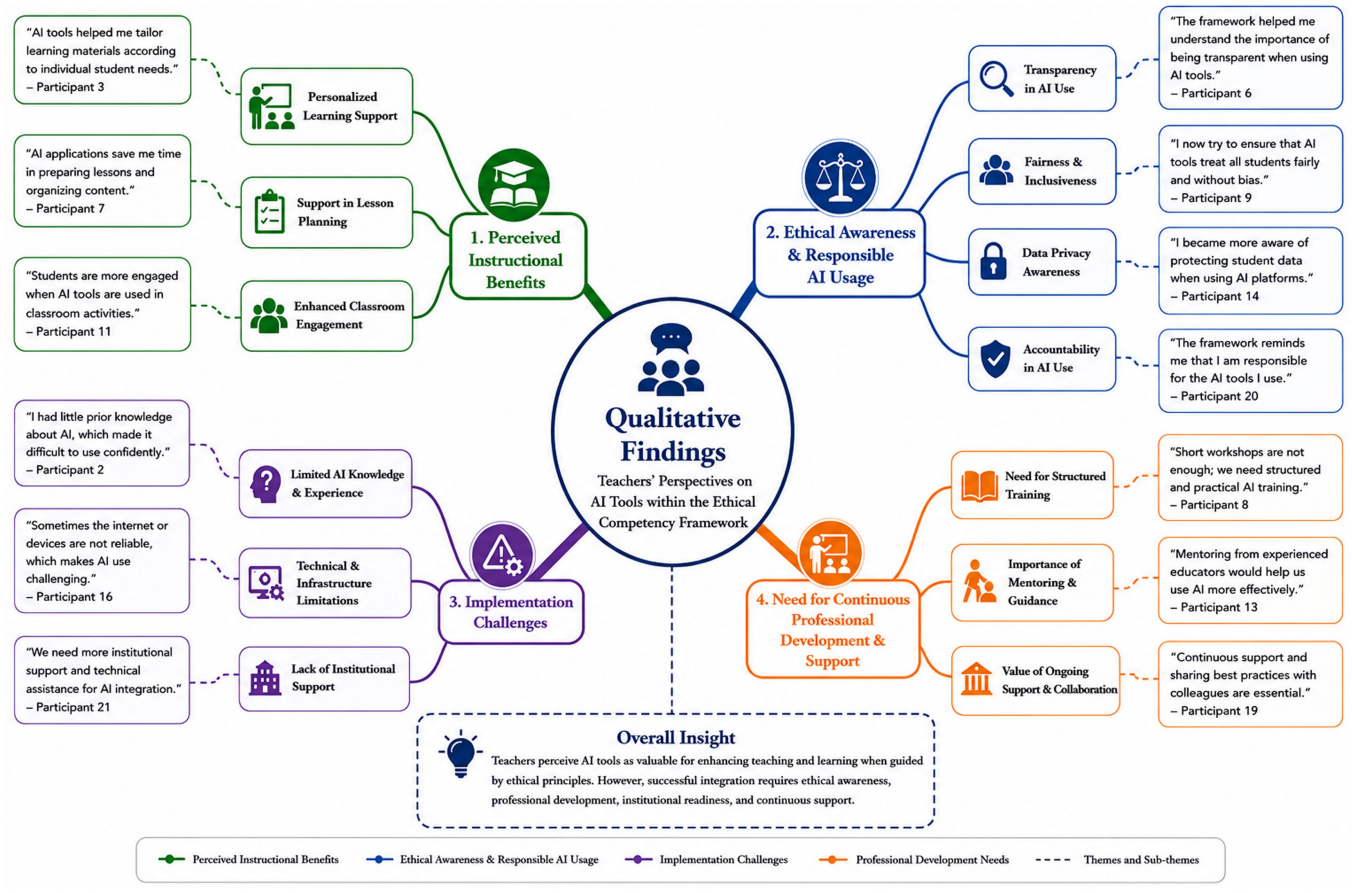
Thematic analysis findings from teacher interviews.

Another major theme involved the need for continuous professional development and institutional support. Many participants emphasized that short-term exposure to AI tools was insufficient for building long-term confidence and competency. Teachers recommended structured training programs, practical workshops, ethical AI guidelines, and ongoing mentoring support to facilitate responsible AI integration within educational environments. Participants further highlighted the importance of collaborative learning opportunities where educators could exchange experiences and best practices regarding AI usage. Figure 7 visually summarizes the key qualitative themes and representative participant responses identified during the thematic analysis process. he qualitative findings suggest that teachers perceived AI technologies as potentially valuable tools for enhancing teaching and learning when implemented within an ethically guided framework. However, the findings also indicate that successful AI integration requires sustained professional development, institutional readiness, ethical governance, and continuous support mechanisms. Given the exploratory nature of the pilot study, these findings should be interpreted as preliminary insights intended to guide future large-scale investigations and framework refinement.

The thematic analysis demonstrates that teachers generally perceived AI technologies positively when implemented within a structured ethical competency framework. However, successful AI integration depends not only on technological accessibility, but also on ethical preparedness, institutional readiness, and continuous professional development support. These findings provide preliminary qualitative evidence supporting the practical relevance of the proposed framework within the educational contexts of Pakistan and Saudi Arabia.

## 5 Discussion

This study aimed to develop an ethical competency framework for the responsible integration of Artificial Intelligence (AI) in education among teachers in Pakistan and Saudi Arabia. The study explored the major ethical challenges associated with AI adoption in educational environments, identified the ethical and practical competencies required for responsible AI usage, and conducted a preliminary pilot evaluation of the proposed framework. The findings suggest that AI technologies have considerable potential to support teaching and learning practices; however, their implementation also introduces significant ethical, pedagogical, and institutional challenges that require careful consideration.

The pilot findings indicate that participating teachers generally perceived AI tools as supportive for improving classroom engagement, instructional planning, personalized learning, and administrative efficiency. These findings are consistent with previous studies highlighting the educational potential of AI-supported technologies in enhancing adaptive learning, instructional effectiveness, and learner engagement (Anggarini et al., 2023; Javaid et al., 2023). At the same time, the findings also revealed growing concerns regarding data privacy, algorithmic bias, transparency, accountability, and equitable access to AI technologies. Similar ethical concerns have been widely discussed in recent AI-in-education literature, particularly regarding the collection and processing of sensitive student information through AI-driven educational systems (Kim and Kwon, 2023; Ng et al., 2023).

The educational environments of Pakistan and Saudi Arabia present additional complexities due to linguistic, cultural, socio-economic, and technological diversity among students and institutions. Ethical AI integration within such contexts requires careful attention to inclusiveness, fairness, accessibility, and contextual adaptability. Previous research has shown that AI systems may unintentionally reinforce educational inequalities when algorithmic models fail to account for diverse learner backgrounds and contextual differences (Jonbekova, 2020; Noble and Dubljević, 2022). Therefore, teacher preparedness becomes a critical factor in ensuring that AI technologies are implemented responsibly and equitably within educational settings.

The findings further emphasize the importance of combining ethical awareness with practical AI integration competencies. Teachers participating in the pilot study reported increased awareness regarding responsible AI practices, particularly in areas related to transparency, fairness, data privacy, and inclusive learning support. However, participants also highlighted the need for stronger practical AI skills to effectively design AI-supported learning experiences, conduct AI-assisted assessments, and facilitate student-centered learning environments. These findings support previous research suggesting that ethical literacy alone is insufficient without practical competency in AI-supported pedagogy and instructional implementation (Holmes et al., 2022; UNESCO, 2024).

An important contribution of this study is the development of a localized ethical competency framework specifically contextualized for Pakistan and Saudi Arabia. Unlike several international AI competency models that primarily emphasize technical AI literacy or general ethical principles, the proposed framework integrates both practical AI integration competencies and ethical governance dimensions within a teacher-centered educational context. The framework combines competencies related to AI-supported instructional design, assessment literacy, student autonomy, inclusiveness, accountability, transparency, and data privacy. This integrated approach contributes to the growing literature on responsible AI adoption in education by addressing both pedagogical and ethical dimensions simultaneously.

The findings also demonstrate similarities and differences between the proposed framework and international AI competency initiatives. For example, Finland’s AI education strategies emphasize inclusiveness, ethical awareness, and digital citizenship, while Singapore’s frameworks focus strongly on accountability, governance, and measurable educational outcomes (Nasir et al., 2024; Younas and Zeng, 2024). China’s AI education initiatives place greater emphasis on AI literacy and practical technological competency development, with comparatively less focus on ethical implementation dimensions (Holmes and Miao, 2023; UNESCO, 2024). In contrast, the framework proposed in this study attempts to balance practical AI integration skills with ethical competencies tailored to the sociocultural and educational realities of Pakistan and Saudi Arabia.

Despite the positive findings, several implementation challenges were identified during the pilot phase. Teachers reported limited prior AI knowledge, lack of institutional training opportunities, insufficient technical support, and concerns regarding ethical governance of AI systems. These findings suggest that successful AI integration in education requires more than technological access alone. Sustainable implementation depends on continuous professional development, institutional readiness, policy guidance, ethical governance mechanisms, and structured teacher training programs. Previous studies similarly emphasize that teacher readiness and institutional support are essential for responsible AI adoption within educational systems (Pedro et al., 2019; Motlagh et al., 2023).

This study has several limitations that should be acknowledged. First, the pilot study involved a relatively small sample size, limiting the generalizability of the findings. Second, the study primarily relied on self-reported teacher perceptions rather than long-term observational or experimental measures of competency development. Third, the implementation period was relatively short and may not fully capture sustained AI integration practices over time. Therefore, the findings should be interpreted as preliminary and exploratory rather than conclusive. Future research should conduct large-scale longitudinal studies involving diverse educational institutions to further validate and refine the proposed framework.

The study highlights the growing importance of ethical and practical AI competencies in contemporary teacher education. The findings suggest that teachers require both ethical awareness and functional AI integration skills to responsibly implement AI technologies within educational environments. By supporting responsible AI usage, promoting inclusiveness, protecting student rights, and strengthening teacher preparedness, the proposed framework may contribute to more ethical, human-centered, and sustainable AI integration practices in education.

## 6 Conclusion

This study developed a contextualized ethical competency framework to support the responsible integration of Artificial Intelligence (AI) in education among teachers in Pakistan and Saudi Arabia. The framework was designed to address major ethical and practical challenges associated with AI adoption in educational environments, including equity and inclusion, student safety, accountability, data privacy, transparency, and responsible AI-supported pedagogy. The proposed framework aligns with internationally recognized AI ethics and educational competency initiatives, including UNESCO’s AI Competency Framework for Teachers and the European Commission’s responsible AI guidelines for education (European Commission, 2022; UNESCO, 2024).

A major contribution of this study is its focus on the sociocultural and educational contexts of Pakistan and Saudi Arabia, where localized ethical AI competency frameworks for teachers remain limited. Unlike several existing international frameworks that primarily emphasize technical AI literacy or general ethical principles, the proposed framework integrates both ethical governance and practical AI integration competencies within a teacher-centered educational perspective.

The pilot study provided preliminary evidence regarding the perceived applicability and usefulness of the proposed framework. The quantitative findings demonstrated moderately positive perceptions regarding effectiveness (M = 3.73), usability (M = 3.63), and ethical compliance (M = 3.83). Qualitative findings further suggested that teachers perceived the framework as supportive for improving ethical awareness, responsible AI usage, and preparedness for AI-supported educational practices. Participants particularly emphasized the importance of transparency, fairness, inclusiveness, and data protection during AI implementation in classrooms.

Despite these encouraging findings, the study also identified several implementation challenges, including limited AI-related knowledge, insufficient technical support, and the need for continuous professional development. These findings suggest that successful AI integration in education requires not only technological access, but also sustained teacher training, institutional readiness, ethical governance mechanisms, and policy-level support.

The study contributes to the growing body of research on ethical AI integration in education by proposing a practical and contextually relevant competency framework for teachers. By emphasizing both ethical awareness and practical AI integration skills, the framework may support more responsible, inclusive, human-centered, and sustainable AI adoption practices within educational systems in Pakistan, Saudi Arabia, and similar developing educational contexts.

### 6.1 Limitations

This study has several limitations that should be acknowledged. First, the pilot study involved a relatively small sample of teachers from Pakistan and Saudi Arabia, which limits the generalizability of the findings across broader educational contexts and subject areas such as science, mathematics, humanities, and social sciences. Therefore, the findings should be interpreted as preliminary and exploratory rather than conclusive.

Second, the study primarily relied on self-reported teacher perceptions regarding the usability, effectiveness, and ethical applicability of AI-supported educational practices. Longitudinal observational or experimental measures of AI competency development were not conducted. Consequently, the study could not fully evaluate the long-term impacts of AI integration on teaching effectiveness, student engagement, learning outcomes, or sustained ethical AI practices within classrooms.

Third, the pilot implementation period was relatively short and may not adequately capture the long-term institutional adoption and integration of AI technologies in educational environments. In addition, contextual factors such as institutional readiness, technological infrastructure, regional educational differences, school characteristics, and variations in teacher digital competency levels across Pakistan and Saudi Arabia were not examined in detail.

Future research should therefore conduct large-scale longitudinal studies involving more diverse educational institutions, educational levels, and teacher populations to further validate and refine the proposed framework. Comparative investigations between urban and rural educational institutions may also provide deeper insights into regional differences in AI adoption and implementation practices. Future studies may further explore the influence of institutional policies, technological accessibility, AI governance mechanisms, and professional development programs on the sustainable and ethical integration of AI in education.

Additionally, future research should investigate the role of AI in supporting inclusive education and students with special educational needs across diverse learning environments. Incorporating student perspectives, classroom observations, and cross-country comparative analyses may provide a more comprehensive understanding of ethical AI adoption, teacher preparedness, and responsible AI integration practices within educational systems.

### 6.2 Policy recommendations

Based on the findings of this study, several policy recommendations are proposed to support the responsible and sustainable integration of Artificial Intelligence (AI) in education within Pakistan and Saudi Arabia.

First, policymakers should develop comprehensive national AI-in-education policies that clearly define ethical standards related to transparency, accountability, fairness, data privacy, inclusiveness, and child safety. These policies should establish guidelines for the responsible use of AI tools within schools, colleges, and higher educational institutions while ensuring alignment with international ethical AI frameworks such as UNESCO’s AI Competency Framework and Responsible AI principles.

Second, governments and educational institutions should invest in digital infrastructure, technological accessibility, and AI-supported educational resources to reduce inequalities in AI adoption across urban and rural educational environments. Adequate technological infrastructure is essential to ensure equitable access to AI-supported learning opportunities for both teachers and students.

Third, continuous professional development programs should be established to strengthen teachers’ practical and ethical AI competencies. Teacher training initiatives should focus not only on technical AI literacy but also on ethical awareness, responsible AI usage, bias mitigation, data protection, transparency, and inclusive pedagogical practices. Structured certification programs and AI competency workshops may further support teachers’ preparedness for AI-integrated educational environments.

Fourth, educational institutions should establish ethical AI governance mechanisms to monitor and evaluate the use of AI technologies in classrooms. Clear institutional guidelines regarding student data usage, AI-assisted assessment practices, algorithmic transparency, and accountability should be implemented to protect student rights and maintain trust in AI-supported educational systems.

Fifth, curriculum developers should integrate AI literacy and ethical AI awareness into teacher education and professional development programs. Future educators should be equipped with both practical AI integration skills and critical understanding of ethical implications associated with AI technologies in educational settings.

Finally, policymakers should encourage collaboration among governments, educational institutions, researchers, technology developers, and industry stakeholders to support the development of contextually relevant and ethically responsible AI solutions for education. Such collaborative initiatives may help ensure that AI technologies positively contribute to learning outcomes, educational equity, teacher support, and sustainable digital transformation within the educational systems of Pakistan and Saudi Arabia.

## Data Availability

The data that support the findings of this study are available from the corresponding author upon reasonable request.
